# Development of Self‐Healing Composite Hydrogel Based on Gelatin and Glycerol Reinforced With Silk Amino Acids and Graphene Oxide

**DOI:** 10.1155/ijbm/4072513

**Published:** 2026-09-23

**Authors:** Chawisorn Samrit, Narongsak Tirasuntarakul, Orapadee Joochim

**Affiliations:** ^1^ Institute of Field Robotics, King Mongkut’s University of Technology Thonburi, Bangkok, Thailand, kmutt.ac.th

**Keywords:** biomaterials, graphene oxide, self-healing, soft robotics

## Abstract

Silk amino acids (SAAs) are widely used in tissue engineering and wound healing due to their biocompatibility and good interaction with biological materials. This study aims to develop a composite hydrogel based on gelatin, deionized water (DI water), and glycerol, with the addition of SAAs to study their effect on self‐healing capability and graphene oxide (GO) to study its effect on the mechanical properties and healing behavior of the material. From tensile testing, when SAA‐containing specimens were cut apart completely and the cut surfaces were brought back into contact and left to heal for 24 h under the specified environmental conditions, the material recovered up to 28.1% of its original tensile strength (the SAAs 20% formulation). For the effect of GO, the addition of GO at 0.5 phr increased the tensile strength by approximately 87% (*p* = 0.0124), while at the same time reducing the self‐healing efficiency to 11.3%–13.4%. The results show that SAA‐containing hydrogels can undergo self‐healing under the specified environmental conditions, although the recovery of mechanical properties after complete cutting remains limited, and the addition of GO reflects a trade‐off between mechanical strength and self‐healing capability. The data obtained from this study can be used as a basis for the development and improvement of self‐healing hydrogels for biomaterial applications, as well as supporting information for further study in soft prosthetic limbs, soft robotic systems, and medical devices in the future.

## 1. Introduction

The evolution of soft robotics and bionic prosthetics has significantly advanced in modern rehabilitation medicine, aiming to restore both functional mobility and natural esthetics [1–3]. Unlike traditional rigid prostheses and rigid body robots [4], soft biomaterials such as hydrogels offer tissue‐like elasticity that effectively mimics the compliance of human skin and muscles [5]. However, a critical challenge remains: These materials are highly susceptible to mechanical damage, such as lacerations from sharp objects, which leads to rapid structural failure and loss of function [6]. The lack of durability and the inability to self‐repair pose significant limitations on the long‐term reliability of these soft prosthetic and soft actuator interfaces.

To address these constraints, researchers have focused on gelatin, a collagen‐derived natural protein polymer that is widely utilized for its exceptional biocompatibility and high cell affinity. Nevertheless, the mechanical strength of gelatin–glycerol alone is often insufficient for load‐bearing prosthetic applications and for use in robotics in unpredictable real‐world environments [7–9]. To introduce advanced functionality, silk amino acids (SAAs) have emerged as highly potent additives. These amino acids derived from silk fibroin facilitate dynamic reversible intermolecular interactions, such as multivalent hydrogen bonding [10]. These interactions serve as the foundation for autonomous self‐healing mechanisms, allowing the polymer network to re‐establish its structural integrity postfracture without external incentive.

Furthermore, to overcome the trade‐off between softness and structural integrity, nanomaterials are increasingly employed as reinforcing agents. Characterized by its outstanding mechanical properties and abundant oxygen‐containing functional groups, GO serves as an ideal nanofiller. It establishes strong interfacial bonds within the gelatin and SAA matrix, forming a robust yet flexible network that enhances tensile strength [10–13].

In this study, a novel self‐healing composite biomaterial was developed, utilizing a gelatin–glycerol matrix integrated with SAAs and reinforced with GO [14]. The primary objective was to evaluate the synergistic effects of these components on mechanical durability and self‐repair efficiency. The integration of silk’s regenerative potential with the structural reinforcement of GO offers a promising approach for the development of durable, biorealistic prosthetic limbs and soft robotic systems, potentially reducing the need for device replacements and ultimately enhancing the quality of life for patients.

## 2. Materials and Methods

### 2.1. Materials

**FIGURE 1:**
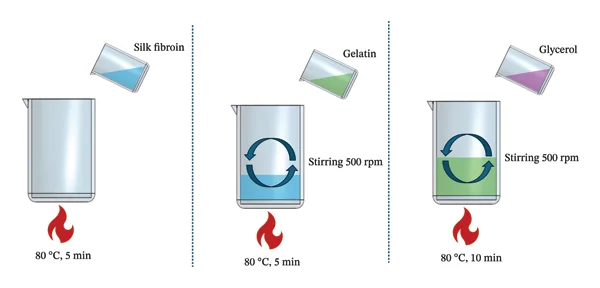
Mixing example of gelatin: glycerol: SAAs.

The materials used in this study consisted of hydrolyzed SAA powder (Vari Silk *p*, VARIATI, Italy), edible bovine‐hide gelatin (INS 428), glycerol (99.85 wt%, INS 422), deionized (DI) water, and graphene oxide (GO) powder (Spec‐G3, EMPOWERMATT Company Limited, Thailand). The formulations were divided into three groups, in which the optimal formulation of each group was used as the starting formulation for the next (Table 1). Group I examined the effect of the glycerol and DI water ratio on the baseline mechanical properties by fixing the gelatin content at 40 parts by weight and varying the gelatin: DI water: glycerol ratio at 40:50:10, 40:40:20, 40:30:30, 40:20:40, 40:10:50, and 40:0:60 by weight. Group II was prepared from the selected base formulation by replacing part of the DI water with an SAA solution, in order to examine the effect of SAA content on the mechanical properties and self‐healing capability. The SAA solution was prepared by dissolving hydrolyzed SAA powder in DI water at SAAs: DI water weight ratios of 10:90, 15:85, and 20:80 (i.e., the SAA powder accounted for 10%, 15%, and 20% by weight of the liquid phase). Group III was prepared from the formulation selected in Group II by adding GO powder to examine the effect of GO on the mechanical properties and self‐healing behavior, with the GO content relative to the liquid phase (SAAs + DI water) set at 0.1:100, 0.5:100, and 1.0:100 by weight. The hydrogels were fabricated by thermal solution casting. For Group I, DI water was first heated to 80 °*C* for 5 min, after which gelatin was added, followed by glycerol, and the mixture was stirred for a further 10 min until homogeneous, with stirring maintained by an overhead stirrer at 500 rpm throughout the process. For Groups II and III, an SAA solution was used to replace part of the pure DI water according to the ratio given above. For Group III only, the GO was dispersed in the liquid phase (DI water–SAAs) using an ultrasonic mixer at 80 W for 30 min to disperse the GO in the liquid phase before mixing with the gelatin [15, 16]. The mixture was then poured into a standard mold. An example of the mixing of gelatin, glycerol, and SAAs during fabrication is shown in Figure 1.

**TABLE 1:** Formulations of the developed hydrogel composites.

Group	Composition	Variable ratios	Key focus
I	Gelatin:DI water: Glycerol	40:10:50, 40:20:40, 40:30:30, 40:40:20, 40:50:10, 40:0:60	Baseline properties
II	Selected group I + SAAs (replacing DI water)	SAAs: DI water = 10:90, 15:85, 20:80	Effect of SAAs on self‐healing
III	Selected group II + GO	GO:(SAAs + DI) = 0.1:100, 0.5:100, 1.0:100	Effect of GO on mechanical and self‐healing behavior

### 2.2. Tensile Properties and Self‐Healing Characterization

**FIGURE 2:**
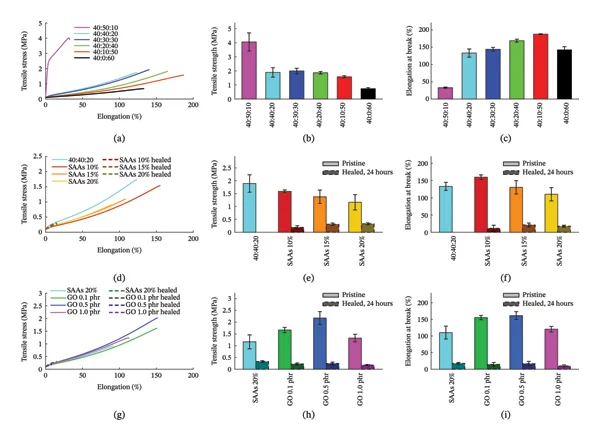
Mechanical properties of the three hydrogel groups: (a) tensile stress vs elongation curves of Group I; (b) mean ± SD of the tensile strength of Group I; (c) mean ± SD of the elongation at break of Group I; (d) tensile stress vs elongation curves of Group II (pristine and healed); (e) mean ± SD of the tensile strength of Group II; (f) mean ± SD of the elongation at break of Group II; (g) tensile stress vs elongation curves of Group III (pristine and healed); (h) mean ± SD of the tensile strength of Group III; (i) mean ± SD of the elongation at break of Group III.

The mechanical properties of the composite hydrogels were evaluated using a universal testing machine (UTM) in accordance with ASTM D412 Method A at a crosshead speed of 500 mm/min, reporting the tensile stress (MPa) and elongation (%). The width and thickness of the narrow section were measured for each cast sheet and entered into the instrument software, which reports the applied load divided by this initial cross‐sectional area. The quantity plotted in Figure 2 and listed in Table 2 is therefore a tensile stress in MPa, and it is the *σ* of equation (1). Variation in thickness between specimens cut from the same sheet appears as scattered in the reported stress and is included in the standard deviations given in Table 2. The initial tensile modulus was taken as the slope of a linear least‐squares fit to the stress vs elongation curve over 1–5% elongation. The tests were divided into three groups according to composition. Group I examined the effect of the weight ratio between gelatin, DI water, and glycerol by pulling the specimens to failure to determine the tensile strength, the elongation at break, and the initial tensile modulus. For Groups II and III, the effects of SAAs and GO on the mechanical properties and self‐healing capability were examined. The specimens of each formulation were divided into two sets: the first set was the pristine specimens, used as the reference; the second set was cut through at the center with a surgical blade, and the surfaces were then brought back into contact without any adhesive and left to heal for 24 h under controlled conditions of 30 °*C* and 60% RH before being tensile‐tested under the same conditions as the pristine specimens. The self‐healing efficiency (*η*) was calculated from recovery of the tensile stress according to the following equation:

(1) η%=σhealedσpristine×100.

**TABLE 2:** Tensile properties and self‐healing efficiency of the hydrogel formulations.

Group	Formulation	Pristine tensile strength (MPa)	Elongation at break (%)	Healed tensile strength (MPa)	*η* (%)
I	40:0:60	0.73 ± 0.08	142.4 ± 9.2	—	—
I	40:10:50	1.57 ± 0.08	187.6 ± 1.0	—	—
I	40:20:40	1.87 ± 0.09	168.9 ± 4.3	—	—
I	40:30:30	1.99 ± 0.19	143.8 ± 5.5	—	—
I	40:40:20	1.89 ± 0.34	133.0 ± 11.8	—	—
I	40:50:10	4.05 ± 0.64	32.7 ± 1.9	—	—
II	SAAs 10%	1.59 ± 0.05	159.8 ± 6.7	0.19 ± 0.06	11.9
II	SAAs 15%	1.38 ± 0.26	130.4 ± 19.3	0.31 ± 0.04	22.6
II	SAAs 20%	1.16 ± 0.30	110.2 ± 19.3	0.33 ± 0.04	28.1
III	GO 0.1 phr	1.67 ± 0.11	155.2 ± 6.6	0.22 ± 0.04	13.4
III	GO 0.5 phr	2.17 ± 0.27	161.3 ± 12.1	0.25 ± 0.06	11.3
III	GO 1.0 phr	1.32 ± 0.16	120.4 ± 8.5	0.18 ± 0.02	13.4

### 2.3. Fourier‐Transform Infrared Spectroscopy (FTIR)

The chemical structure of the composite hydrogels was analyzed by FTIR using a Thermo Scientific Nicolet spectrometer in attenuated total reflectance (ATR) mode with a diamond crystal (Smart iTX accessory), without any additional sample preparation. Spectra were collected over the wavenumber range of 400–4000 *c* *m* ^−1^ at a resolution of 4 *c* *m* ^−1^ with atmospheric suppression. The FTIR analysis was carried out to (1) confirm the presence of SAAs and glycerol within the gelatin matrix and (2) evaluate changes in the structure. In addition, to examine the reconnection of the bonding network at the healed region, the spectra of samples in three states were compared: the pristine sample, the freshly cut surface, and the surface after being brought into contact and left to self‐heal for 24 h (healed) under controlled conditions of 30°C and 60% RH.

### 2.4. X‐Ray Diffraction (XRD)

The crystalline structure and molecular arrangement of the composite hydrogels were analyzed by XRD using a Rigaku diffractometer with Cu Kα radiation (*λ* = 1.5406 *Å*), operated at 40 kV and 30 mA. The data were collected over a 2*θ* range of 5 − 80° with a step size of 0.01° and a scan rate of 6.0 deg/min. The XRD analysis was carried out to evaluate changes in the average interchain spacing of the gelatin matrix upon the addition of glycerol and SAAs, based on the position of the amorphous halo. The interplanar spacing (d‐spacing) was calculated using Bragg’s equation (*n* *λ* = 2*d* *·*sin*θ*).

### 2.5. Statistical Analysis

Each formulation was tested in triplicate (*n* = 3), and the results are reported as the mean ± standard deviation (mean ± SD), shown as error bars in all mechanical‐testing graphs. Statistical analysis was performed using MATLAB (R2024b, The MathWorks, Inc., USA). Differences between formulations were compared by one‐way analysis of variance (ANOVA) followed by Tukey’s honestly significant difference (HSD) post hoc test, and the pristine and healed specimens were compared using Welch’s *t*‐test, as the two sets were different specimens with unequal variances. Statistical significance was set at *p* < 0.05.

### 2.6. Material Interface Healing and Incision Recovery Analysis

**FIGURE 3:**
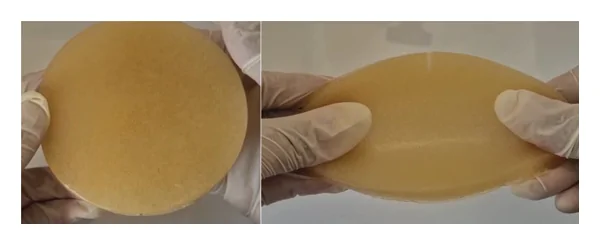
Group II material that has been cut and healed.

**FIGURE 4:**
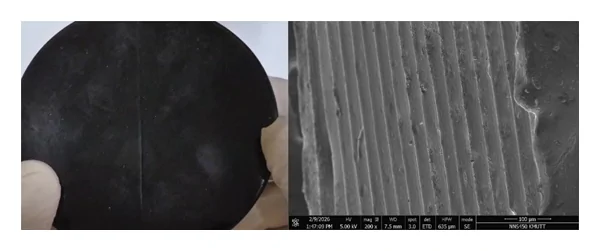
Group III material that has been healed and SEM photographs of the cut.

To evaluate the self‐repairing performance of Group II and Group III composite hydrogels, a controlled incision recovery test was conducted. Unlike total fracture tests, this method involved creating a partial transection to simulate common physical damage, such as superficial lacerations or incised wounds encountered in practical prosthetic applications [17, 18]. A surgical scalpel was utilized to create a precise longitudinal incision on the hydrogel samples within the Petri dish. The depth of the incision was controlled at two‐thirds (2/3) of the total material thickness. The remaining one‐third (1/3) of the base acted as a structural “bridge,” ensuring precise alignment and constant physical contact between the fractured interfaces without the need for external fixation. This experimental setup effectively mimics a standard “incision” injury, like a paper cut or a shallow blade wound. Following the incision, the specimens were maintained at 30°C and 60% RH. The recovery process was allowed to proceed autonomously for a duration of 24 h before the samples were subjected to visual inspection and further mechanical verification to assess interfacial healed efficiency. The cut and healed Group II specimens are shown in Figure 3, and the healed Group III specimens together with SEM images of the cut surface in Figure 4.

## 3. Results and Discussion

### 3.1. Mechanical Properties of Material (Group I)

The ratio between glycerol and DI water had a clear effect on the mechanical properties of the hydrogels. The tensile results of all six formulations are shown in Table 2. One‐way ANOVA revealed significant differences in both the tensile strength (*F* = 37.28, *p* < 0.001) and the elongation at break (*F* = 188.93, *p*<0.001).

The ratio between glycerol and DI water affected the mechanical properties of the hydrogels, as shown in Table 2. One‐way ANOVA revealed significant differences in both the tensile strength (*F* = 37.28, *p*<0.001) and the elongation at break (*F* = 188.93, *p*<0.001). Tukey’s HSD post hoc test showed that the tensile strengths of the 40:10:50, 40:20:40, 40:30:30, and 40:40:20 formulations were not significantly different from one another (*p* ≥ 0.5940 for all pairwise comparisons). When the glycerol content was between 20 and 50 parts, the tensile strength reached a comparable level. Clear differences appeared only at the two ends of the range: the 40:0:60 formulation gave the lowest value, and the 40:50:10 formulation the highest. In contrast, the initial tensile modulus increased steadily as the glycerol fraction decreased, from 0.341 ± 0.002 MPa for the 40:0:60 formulation to 43.59 ± 3.83 MPa for the 40:50:10 formulation, consistent with the role of glycerol as a plasticizer, with the glycerol distributed between the gelatin chains reducing interchain attraction. The base formulation for Group II was therefore not selected primarily based on the tensile strength since the mid‐range formulations were not statistically different, but on two criteria together. First, the selected formulation had to have a modulus high enough for the specimens to retain their shape after molding for use as a soft robot. Second, it had to contain enough water to dissolve the SAA powder in the subsequent step. The 40:40:20 formulation satisfied both criteria, providing a modulus of 2.33 ± 0.81 MPa, distinctly higher than that of the more glycerol‐rich formulations while still containing 40 parts of water for preparing the SAAs solution. Although the 40:50:10 formulation gave the highest tensile strength, its elongation was only 32.7% and its modulus reached 43.59 MPa, which is overly stiff for soft‐material applications. Moreover, it contained merely 10 parts of water, an amount insufficient to dissolve the SAAs.

### 3.2. Effect of SAAs on Self‐Healing (Group II)

The tensile results of the Group II hydrogels are shown in Table 2 (Group II). One‐way ANOVA of the pristine specimens across the three formulations found no significant difference (*F* = 2.56, *p* = 0.1568), and comparing the non‐SAA base formulation with the 10% SAA formulation by Welch’s *t*‐test found no significant difference (*t* = 1.54, *p* = 0.2576), indicating that the addition of SAAs did not reinforce the matrix. However, the SAAs played an important role in self‐healing capability. When the cut surfaces were brought back into contact and left to heal for 24 h, the SAA‐containing specimens were able to bear a measurable tensile load, with *η* ranging from 11.9% to 28.1%. Comparing the pristine and healed specimens of the 10% SAA formulation, Welch’s *t*‐test revealed a significant difference (*t* = 29.10, *p*<0.001). The healed interface remained a distinctly weaker region than the bulk material. One notable observation is that although the tensile strength after healing recovered to only 11.9%–28.1%, the initial tensile modulus of the healed specimens was close to that of the pristine specimens. In the 10% SAA formulation, for example, the moduli of the pristine and healed specimens were 1.08 and 1.02 MPa, respectively. This indicates that the healed interface can transmit load at low strain no differently from the bulk material but fractures at a much lower strain (approximately 10%–20%) than the pristine specimens. The limitation of the healed interface lies in its capacity to withstand high strain, not in its stiffness.

### 3.3. Effects of GO (Group III)

The tensile results of the Group III hydrogels, prepared from the SAA 20% base formulation, are shown in Table 2 (Group III). One‐way ANOVA of the pristine specimens across the three GO loadings revealed a significant difference (*F* = 14.61, *p* = 0.0049), and Tukey’s HSD test showed that the 0.5 phr formulation differed significantly from both the 0.1 phr (*p* = 0.0420) and the 1.0 phr (*p* = 0.0041) formulations. The addition of GO at 0.5 phr increased the tensile strength from 1.16 ± 0.30 MPa (the GO‐free formulation) to 2.17 ± 0.27 MPa, an increase of approximately 87%, together with an increase in the elongation at break from 110.2% to 161.3%. In contrast, the 0.1 phr and 1.0 phr formulations were not significantly different from the SAAs 20% formulation (*p* = 0.0853 and *p* = 0.4749). The response was not monotonic with GO content. XRD of the same formulations (Section 3.4) shows no GO reflection at any loading, so this behavior is not attributed to agglomeration on the present evidence. Another significant result is that the addition of GO reduced the self‐healing efficiency at all concentrations studied, from 28.1% in the GO‐free formulation to 11.3–13.4%.

### 3.4. Physicochemical Characterization of the Gelatin–SAA–GO Composites

**FIGURE 5:**
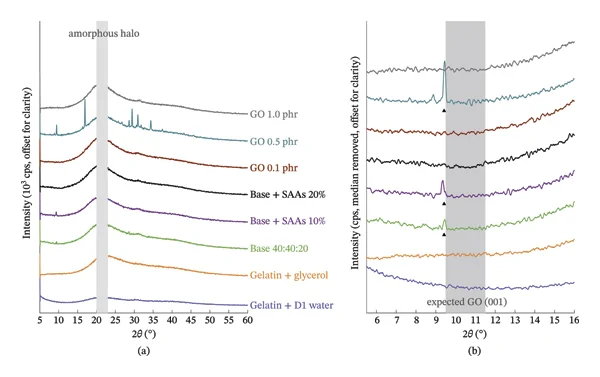
(a) FTIR‐ATR spectra and (b) XRD patterns of the eight formulations. Each spectrum in (a) is the mean spectrum of three scans, converted to absorbance and normalized individually, so band positions rather than intensities are comparable between traces. Traces in both panels are offset vertically and follow the same order and color, from bottom to top: gelatin + DI water, gelatin + glycerol, 40:40:20, SAAs 10%, SAAs 20% without GO, and the SAAs 20% base with 0.1, 0.5, and 1.0 phr GO. Dotted lines in (a) mark the assigned band positions; the shaded band in (b) marks the amorphous halo of gelatin at 20 − 23°2*θ*. The narrow peaks in the 0.5 phr and SAAs 20% patterns come from stray crystalline grains (Section 3.4).

Every spectrum contained the bands of a protein system: amide A at 3280–3289 *c* *m* ^−1^, C–H at 2938–2962 *c* *m* ^−1^, amide I at 1629–1644 *c* *m* ^−1^, amide II at 1548–1552 *c* *m* ^−1^, and C–O at 1032–1039 *c* *m* ^−1^. Every pattern was that of a semi‐amorphous solid, with one broad halo between 20 and 23°2*θ* and no sharp reflection belonging to the formulation. Glycerol, SAAs up to 20% of the liquid phase, and GO up to 1.0 phr all enter the network without forming a crystalline phase. The amide I maximum lies at 1629 *c* *m* ^−1^ for the sample made with water alone and at 1644 *c* *m* ^−1^ for the sample made with glycerol alone, a shift of about four times the spectral resolution. Their difference spectrum shows a loss below 1620 *c* *m* ^−1^ and a gain above 1640 *c* *m* ^−1^, so intensity moves from the part of the amide I band where hydrogen‐bonded and aggregated structures absorb to the part where turns and disordered segments absorb [19]. Diffraction answers the other half of the question. The glycerol‐only sample gave the smallest interchain spacing of the eight, 3.9*Å*, against 4.1*Å* for the water‐only sample and for the base formulation. The halo position is method‐dependent to about 0.1*Å*, so only the 0.2*Å* glycerol‐to‐water difference is read. Glycerol does not push the chains apart; water does. Its effect on the modulus in Section 3.1 is a change in the secondary structure, not in chain separation. SAAs cannot be separated by elemental composition, so difference spectroscopy was used. Subtracting the base spectrum from the SAA‐containing spectra, with both scaled to the amide I maximum, leaves a positive band at 1601 *c* *m* ^−1^, in the region of asymmetric carboxylate stretching, a group carried by the free amino acids and not by gelatin [20]. It grows from 0.12 at 10% SAAs to 0.14 at SAAs 20%. Water cannot account for it: the total liquid is the same in all three formulations, and raising the SAA fraction from 0% to 20% lowers the water from 40 to 32 parts while the band grows. Diffraction showed no change over the same series; the base and the 10% SAA formulation differing by 0.05*Å*. Neither method identifies the GO directly. At 0.1–1.0 phr of the liquid phase, GO is 0.04–0.4 wt% of the whole formulation. The second derivative of the GO‐containing spectra locates no band between 900 and 1800 *c* *m* ^−1^ that is absent from the GO‐free formulation. No reflection was found between 9.5 and 11.5°2*θ*, where stacked GO gives its (001) reflection, and the halo stayed at 4.2 Å at all four loadings [21, 22]. Narrow peaks near 9.4°2*θ* are not from the GO: the same feature appears in the GO‐free base formulation and in the base with 10% SAAs, and all such peaks are below 0.2° wide, at the angular resolution of the instrument, while stacked GO gives a reflection one to two degrees wide. They come from stray crystalline grains. The absence of the (001) reflection needs more careful reading than a detection limit alone: that reflection reports the repeat distance between stacked sheets, so an exfoliated sheet gives none at any concentration, and its absence is evidence about stacking rather than about amount. The GO is present. At 0.5 phr, it is 0.20 wt% of the formulation, a volume fraction of 1.2 × 10^−3^, which for a sheet of 0.8‐nm thick places the sheets of the order of 700 nm apart across an interface of the order of 3 m^2^ per gram of hydrogel. Reinforcement depends on that interface, which scales with exposed sheet area rather than with mass [11], so the significant reinforcement at 0.5 phr is itself indirect evidence that most of the GO is dispersed. The loss at 1.0 phr is consistent with a loss of dispersion and with interference with the SAA hydrogen‐bonding network alike, and the present measurements do not separate them. The healed interface of the pristine material, a freshly cut surface, and a cut surface healed for 24 h were compared for the five formulations whose cut faces carry a load, by root‐mean‐square difference over 1500–1700 *c* *m* ^−1^ after scaling to the amide I maximum. Among the three states, the difference was 0.004–0.041, against 0.064 between two formulations of different composition and 0.002 between two scans of one specimen. In every formulation, the three states lie closer to each other than two formulations do. Twenty‐four hours after the cut, the healed region is not chemically distinguishable from the bulk. Read with Section 3.2, where healed specimens kept the modulus of the pristine material but failed at much lower strain, this places the limitation of the healed interface in the strain it can carry, not in the chemistry it reforms. The chemical structure and the molecular arrangement were measured on the same eight formulations by FTIR‐ATR and by XRD (Figure 5).

## 4. Application of Materials

**FIGURE 6:**
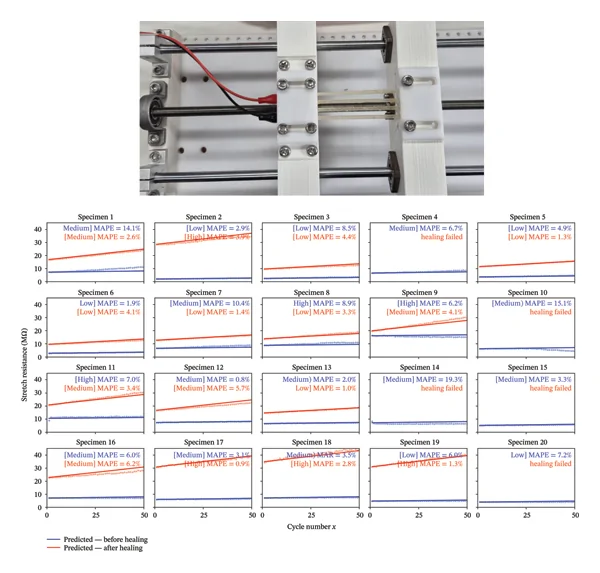
Appearance of a specimen mounted on the test rig, together with the stretch‐and‐release results for all 20 specimens, each shown with the fitted mean trend line. For specimens in which healing was unsuccessful, only the blue curve is present.

To evaluate the practical applicability of the material, we prepared a demonstration of tensile strain sensors. For the sensor demonstration, we selected the formulation with the best healing performance, the SAA 20% group. Twenty sensor specimens were molded, each with overall dimensions of 20 mm in width, 85 mm in length, and 0.5 mm in thickness. The hydrogel was cast into a prepared PLA mold and left to set for 12 h before being mounted on the test rig. The test consisted of repeated stretch‐and‐release cycles driven by a custom rig built from a stepper motor and a leadscrew, running 50 cycles at a displacement of 10 mm. Resistance was measured with a voltage‐divider circuit using a 10‐MΩ reference resistor and a 5‐V supply. After completing this test, we cut each specimen completely through, allowed it to heal for 24 h, remounted it on the same rig, and repeated the identical test once. All 20 specimens completed the 50 cycles before healing. After the cut and 24 h of healing, 15 of the 20 completed the same test. Before healing, the specimens gave a resistance of 6.30 ± 2.89 MΩ; after healing, the resistance rose to 21.01 ± 8.82 MΩ. During the posthealing cycling, the resistance of the specimens increased with every stretch, and this increase follows a linear relationship,

(2) Rx=mx+R0,

where *R*_0_ is the resistance measured on the first cycle at full extension. The specimen mounted on the test rig and the stretch‐and‐release results for all 20 specimens are shown in Figure 6.

**FIGURE 7:**
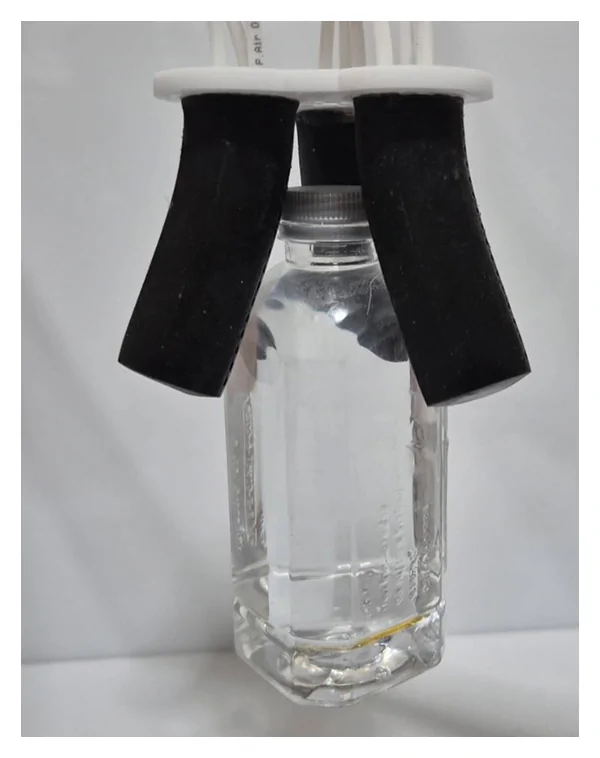
Pneumatic soft gripper molded from the Group III formulation, holding a plastic bottle of 370 g, the largest mass lifted without slipping.

The material was also molded into a pneumatic soft gripper from the 0.5 phr GO formulation, the strongest of the series. The finger takes its form from the flagellar and ciliary driving principle described by Miao et al. [23], in which a tubular chamber bends as it inflates. Air supplied at 0.16 MPa to the chamber inside each finger closes the gripper onto an object. Closed on a plastic bottle, it lifted and held a maximum mass of 370 g without slipping (Figure 7). Gripping force was not measured separately from friction at the contact surface, so the result is reported as load‐bearing capacity only.

## 5. Conclusions

A self‐healing composite hydrogel of gelatin, glycerol, SAA, and GO was prepared, and its mechanical properties, chemical structure, and healing behavior were measured. The ratio of glycerol to DI water set the properties of the matrix. The initial tensile modulus rose as the glycerol fraction fell from 0.341 ± 0.002 MPa at 40:0:60 to 43.59 ± 3.83 MPa at 40:50:10, while the tensile strength of the mid‐range formulations did not differ. The 40:40:20 formulation was carried forward because it held its shape after molding, with a modulus of 2.33 ± 0.81 MPa, and still contained enough water to dissolve the SAAs. FTIR and XRD account for the role of glycerol: the amide I band shifts by 15 cm^−1^ toward the disordered side, while the interchain spacing falls rather than rises, so glycerol acts on the secondary structure of the protein and not on the separation between chains. SAAs did not change the tensile strength (*F* = 2.56, *p* = 0.1568), but they allowed the cut faces to carry a load once returned to contact, with a healing efficiency of 11.9–28.1%, the highest value at SAAs 20%. Their incorporation was confirmed by a carboxylate band at 1601 cm^−1^ that grows with the SAA content while the water content falls. Healed specimens kept the modulus of the pristine material, 1.02 against 1.08 MPa at 10% SAAs, and FTIR found no chemical difference between the healed region and the bulk in any of the five formulations tested. What the healed interface loses is the strain it can carry, not the bonding it reforms. GO gave a trade‐off. At 0.5 phr, the tensile strength rose from 1.16 ± 0.30 to 2.17 ± 0.27 MPa, about 87% (*p* = 0.0124), and the elongation at break from 110.2% to 161.3%, while the healing efficiency fell to between 11.3% and 13.4% at every loading. The response was not monotonic with the GO content. Neither FTIR nor XRD identifies the GO directly: no infrared band and no diffraction peak were found at any loading. The absent (001) reflection reports the absence of stacking rather than the absence of material, since an exfoliated sheet gives no reflection at any concentration. A strain‐sensing demonstration gave a practical measure of the healing: after being cut through and healed for 24 h, 15 of 20 specimens still responded to 50 stretch‐and‐release cycles.

Three limits apply to these results. Healing was measured after one cut, so behavior over repeated cut‐and‐heal cycles is unknown. All specimens were healed and tested at 30°C and 60% RH, so the response of a gelatin‐based hydrogel to other temperatures and humidities has still to be measured. The dispersion state of the GO is below the detection limit of the methods used and remains open [24]. The healing efficiency reached here is not enough for a structural material in a load‐bearing actuator. The value of the work is baseline data for a hydrogel whose matrix is made entirely from natural materials, which rejoins a cut surface at room temperature with no chemical crosslinker and no external stimulus, and as a starting point for self‐healing hydrogels in biomaterials, soft prosthetic limbs, soft robotic systems, and medical devices.

## Author Contributions

Chawisorn Samrit: conceptualization, methodology, investigation, formal analysis, and writing–original draft. Narongsak Tirasuntarakul: data curation, visualization, validation, and writing–review and editing. Orapadee Joochim: validation, formal analysis (literature review), and writing–review and editing.

## Funding

This work was supported by the Thailand Science Research and Innovation through the Fundamental Fund.

## Conflicts of Interest

The authors declare no conflicts of interest.

## Data Availability

Data are available from the corresponding author upon request.
